# Lipocalin Prostaglandin D Synthase and PPARγ2 Coordinate to Regulate Carbohydrate and Lipid Metabolism In Vivo

**DOI:** 10.1371/journal.pone.0039512

**Published:** 2012-07-05

**Authors:** Sam Virtue, Mojgan Masoodi, Vidya Velagapudi, Chong Yew Tan, Martin Dale, Tapani Suorti, Marc Slawik, Margaret Blount, Keith Burling, Mark Campbell, Naomi Eguchi, Gema Medina-Gomez, Jaswinder K. Sethi, Matej Orešič, Yoshihiro Urade, Julian L. Griffin, Antonio Vidal-Puig

**Affiliations:** 1 University of Cambridge Metabolic Research Laboratories, Institute of Metabolic Science, Cambridge, United Kingdom; 2 Elsie Widdowson Laboratory, Medical Research Council Human Nutrition Research, Cambridge, United Kingdom; 3 VTT Technical Research Centre of Finland, Espoo, Finland; 4 Department of Medicine Innenstadt, Endocrinology/Diabetes University Hospital, Munich, Germany; 5 Osaka Bioscience Institute, Osaka, Japan; 6 Departamento de Bioquímica, Fisiología y Genética Molecular, Universidad Rey Juan Carlos, Madrid, Spain; 7 Department of Biochemistry, Medical Research Council Human Nutrition Research, Cambridge, United Kingdom; University of Las Palmas de Gran Canaria, Spain

## Abstract

Mice lacking Peroxisome Proliferator-Activated Receptor γ2 (PPARγ2) have unexpectedly normal glucose tolerance and mild insulin resistance. Mice lacking PPARγ2 were found to have elevated levels of Lipocalin prostaglandin D synthase (L-PGDS) expression in BAT and subcutaneous white adipose tissue (WAT). To determine if induction of L-PGDS was compensating for a lack of PPARγ2, we crossed L-PGDS KO mice to PPARγ2 KO mice to generate Double Knock Out mice (DKO). Using DKO mice we demonstrated a requirement of L-PGDS for maintenance of subcutaneous WAT (scWAT) function. In scWAT, DKO mice had reduced expression of thermogenic genes, the *de novo* lipogenic program and the lipases ATGL and HSL. Despite the reduction in markers of lipolysis in scWAT, DKO mice had a normal metabolic rate and elevated serum FFA levels compared to L-PGDS KO alone. Analysis of intra-abdominal white adipose tissue (epididymal WAT) showed elevated expression of mRNA and protein markers of lipolysis in DKO mice, suggesting that DKO mice may become more reliant on intra-abdominal WAT to supply lipid for oxidation. This switch in depot utilisation from subcutaneous to epididymal white adipose tissue was associated with a worsening of whole organism metabolic function, with DKO mice being glucose intolerant, and having elevated serum triglyceride levels compared to any other genotype. Overall, L-PGDS and PPARγ2 coordinate to regulate carbohydrate and lipid metabolism.

## Introduction

We have previously described the phenotype of the Peroxisome Proliferator Activated Receptor γ2 KO mouse on a sv129 background (sv129 PPARγ2 KO mice) [1], a mouse model notable for its surprisingly mild phenotype in terms of adipose tissue function and insulin resistance. The nuclear receptor Peroxisome Proliferator Activated Receptor γ (PPARγ*)* is essential for adipogenesis [2] and has two isoforms, PPARγ1 and PPARγ2. *In vivo* PPARγ1 has a broad tissue distribution whereas, under normal physiological conditions, PPARγ2 expression is almost exclusive to adipose tissue [3].

In white and brown adipose tissue, approximately half of all PPARγ protein and PPARγ transcript is PPARγ2 [3]. Furthermore, PPARγ2 has a higher transcriptional activity than PPARγ1 [4], suggesting that loss of PPARγ2 from adipose tissue would represent a loss of the majority of PPARγ mediated transcriptional function. In accordance with these data, two separate studies demonstrated that when either preadipocytes or mouse embryonic fibroblasts (MEFS) were obtained from PPARγ2 KO mice, they had a greatly impaired capacity for adipogenesis *in vitro* when compared to wild-type cells [5], [6].

While much focus on PPARγ function has been directed towards its adipogenic role and its control of glucose homeostasis, PPARγ also regulates many of the enzymes involved in lipid uptake, synthesis and release. Mice and humans with dominant negative mutations in *PPARγ* have severe impairments in lipid clearance [7], [8], pointing to an important role for PPARγ in daily lipid buffering. The finding that mice harbouring a P465L Dominant Negative (DN) mutation in the *PPARγ* gene actually had normal glucose tolerance but impaired lipid clearance suggests that PPARγ may be more crucial for lipid homeostasis than carbohydrate metabolism [7]. *PPARγ2* KO mice also exhibited almost normal glucose tolerance and insulin sensitivity but also had severe impairments in lipid clearance compared to wild type controls.

A further layer of complexity regarding the regulation of lipid buffering comes from the fact that not all adipose tissue depots contribute equally to fatty acid uptake and release. Different depots have different rates of lipid release and uptake [9] and the size of different adipose tissue depots has been associated with insulin resistance. Subcutaneous adipose tissue mass has a neutral, or in the case of gluteofemoral adipose tissue, even a positive association with insulin sensitivity. Conversely, intra-abdominal fat mass is strongly associated with insulin resistance [10], [11]. Interestingly both P465L DN and PPARγ2 KO mice exhibited alterations in adipose tissue distribution with P465L DN mice having an increased subcutaneous to epididymal ratio [12] and PPARγ2 KO mice having reduced intra-dermal lipid levels [5].

We and others have put forward the adipose tissue expandability hypothesis to suggest how obesity leads to metabolic complications. Essentially, a loss of adipose tissue capacity to store lipid leads to the accumulation of lipids in non-adipose tissues. As lipids accumulate ectopically, they cause toxic insults to these tissues resulting in pathologies such as insulin resistance [13], [14]. Based on the adipose tissue expandability hypothesis we expected mice lacking PPARγ2 to have severe impairments in insulin sensitivity. The impairments in *in vitro* adipogenesis in *PPARγ2* null preadipocytes and the known roles for PPARγ in lipid handling suggested that adipose tissue would be compromised in its ability to store lipid. Given the unexpectedly normal phenotype of the *PPARγ2* KO mouse we hypothesised that there must be mechanisms acting *in vivo* to protect *PPARγ2* null animals from developing severe metabolic disturbances. To try to identify possible compensatory mechanisms we used microarray analysis to compare white and brown adipose tissue from WT and *PPARγ2* KO mice. From these microarray analyses we identified Lipocalin prostaglandin D synthase (L-PGDS).

L-PGDS is a bi-functional molecule capable of synthesising D series prostaglandins and acting as a carrier of small lipophilic molecules [15]. In humans, serum L-PGDS levels have been positively associated with the severity of coronary artery disease [16], whereas in mice ablation of L-PGDS has been reported to aggravate the development of atherosclerosis [17]. However, its involvement in insulin sensitivity and glucose tolerance is less clear [17], [18]. Furthermore, in humans *L-PGDS* expression is higher in scWAT than abdominal WAT [19], suggesting it may modulate depot-specific differences in adipose tissue function.

To determine if L-PGDS was able to protect mice lacking PPARγ2 from impairments in metabolism we generated a double knock-out mouse lacking both PPARγ2 and L-PGDS (DKO). Removing L-PGDS from PPARγ2 KO caused glucose intolerance and resulted in dyslipidemia.

## Materials and Methods

### Generation of Mice Homozygous for PPARγ2 KO and L-PGDS KO

Mice heterozygous for a disruption in exon B1 of *PPARγ2* on a C57Bl/6J background (*PPARγ2* ^+/−^) were crossed with mice heterozygous for a disruption in exons II–V in the *L-PGDS* gene [20] on a C57Bl/6J background to obtain mice heterozygous for both the *PPARγ2* and *L-PGDS* (*PPARγ2* ^+/−^ L-*PGDS* ^+/−^). These mice were crossed to obtain the four experimental genotypes: WT (*PPARγ2* ^+/+^ *L-PGDS* ^+/+^), *PPARγ2* KO (*PPARγ2* ^−/−^ *L-PGDS* ^+/+^), *L-PGDS* KO (*PPARγ2* ^+/+^ *L-PGDS* ^−/−^) and DKO (*PPARγ2* ^−/−^ *L-PGDS* ^−/−^). Mice were phenotyped on C57Bl/6J background (at least 8 generations). DKO mice were born at the expected Mendelian ratios (908 mice, Chi squared  = 0.22). Genotyping for deletion of *PPARγ2* or *L-PGDS* was performed by PCR using previously described protocols [5], [20].

### Animal Care and Diets

Male mice were housed at a density of four animals per cage in a temperature-controlled room (20–22°C) with 12-h light/dark cycles. Food and water were available *ad libitum* unless noted. All animal protocols used in this study were approved by the UK Home Office (License no. 80/2098) and the University of Cambridge. Animals were fed on a normal chow diet (10% of calories derived from fat; D12450B, Research Diets).

### Blood Biochemistry

Enzymatic assay kits were used for determination of plasma FFAs (Roche) and triglycerides (Sigma-Aldrich, St. Louis, Missouri, USA). Elisa kits were used for measurements of Insulin (DRG Diagnostics International Limited) according to manufacturer’s instructions. Serum biochemistry was analysed in male C57Bl/6 mice of 4 months of age, n = 8 per group.

### Hepatic Triglyceride Analysis

Lipids were extracted from 100 mg of homogenised liver by heating to 85°C in 1 ml of 5% NP-40 in water. Triglyceride levels in extracts were assessed by enzymatic assay (Biovision) according to manufacturers instructions. Triglyceride content was analyzed in male C57Bl/6 mice of 5 months of age, n = 8 per group.

### Oxygen Consumption (VO2) and Respiratory Exchange Ratio (RER) from Indirect Calorimetry

Animals were placed in a Comprehensive laboratory animal monitoring system (Columbus Instruments, Ohio, USA) attached to a custom-built oxygen and carbon dioxide monitoring system. The system was maintained at 22°C. Oxygen consumption (VO_2_) and respiratory exchange ratio (RER) (VCO_2_/VO_2_) were measured in mice at ages as described in figures. Oxygen consumption was measured over a 48–72 hour period using a custom built oxygen and carbon dioxide monitoring system. Airflow rates were 400 ml/min and measurements of oxygen concentration and carbon dioxide concentration in room air and air leaving each cage were measured every 18 minutes [21]. Indirect calorimetry was performed on male C57Bl/6 mice of 4 months of age, n = 8 per group.

### Insulin Tolerance Test (ITT)

Mice were fasted for 6 hours from 8 am until 2 pm. Mice were injected intraperitoneally with 0.75 U/Kg of human insulin (Actrapid, Novo Nordisk). Blood glucose levels were measured using a one touch ultra glucose meter (Lifescan, CA, USA) [22]. Insulin tolerance tests performed on male C57Bl/6 mice of 4.5 months of age, n = 8 per group.

### Glucose Tolerance Tests (GTT)

Mice were fasted overnight from 4 pm until 9 am the next day. Mice were injected intraperitoneally with glucose (1 g/kg) and blood glucose levels were measured using a one touch ultra glucose meter (Lifescan, CA, USA) [22]. Glucose tolerance tests performed on male C57Bl./6 mice of 4 months of age, n = 8 per group.

### Histology

Samples for histology were placed in 10% buffered formalin overnight before transfer to 70% ethanol and later embedding in paraffin. Multiple sections were stained with haematoxylin and eosin for morphological analysis. Percentage lipid content was determined by morphometric analysis by measuring the proportion of white area (lipid) per slide. The average lipid area in BAT per mouse was calculated from at least 3 separate sections. Analysis was carried out using CELLˆP analysis software (Olympus, Southend-on-Sea, UK). BAT histology performed at 4 months, liver histology at 5 months. All; male C57Bl/6 mice, n = 8 per group.

### Q-RT-PCR

RNA was isolated from ground tissues using STAT-60 reagent (TEL-TEST) followed by chloroform extraction and isopropanol precipitation. Complimentary DNA was generated from 500 ng of RNA using M-MLV reverse transcriptase and master mix (Promega) in a 20 µl reaction with 2.5 mM MgCl_2_, 1.25 mM dNTPs and 5 µg/ml random hexamers at 37°C for 1 hour. cDNA was diluted 75 fold and 5 µl of diluted cDNA was used in a 12 µl real time PCR (RT-PCR) reaction using TaqMan primers and probes or SYBR green reagent (Applied Biosystems) according to manufacturers instructions. Reactions were run in duplicate for each sample and quantified in the ABI Prism 7900 sequence detection system (Applied Biosystems). Data was normalised to 18s rRNA. Primer sequences available on request. eWAT, scWAT and BAT analyzed at 4 months, liver at 5 months. All; male C57Bl/6 mice, n = 8 per group.

### Western Blotting

All western blots were carried out using 10% polyacrylamide gels (Biorad). 20 ug of protein was used for all samples. All primary antibodies from Cell Signalling (catalogue number in brackets) pHSL S600 (#4216), pHSL S565 (#4137), tHSL(#4107), ATGL (#2138), pAMPKα T172 (#2535) and tAMPKa (#2532). All antibodies used at a 1∶1000 dilution in 5% BSA. Quantification was carried out using ImageJ (NIH). All; male C57Bl/6 mice, 4 months of age n = 6 per group except scWAT ATGL, n  = 3.

### Eicosanoid Extraction and Quantification

Lipidomic analysis of lipid mediators in brown adipose tissue was performed according to our published protocol [23]. In summary: Brown adipose tissue from WT (PPARγ2^+/+^ L-PGDS ^+/+^), PPARγ2 KO (PPARγ2^−/−^ L-PGDS ^+/+^), L-PGDS KO (PPARγ2^+/+^ L-PGDS ^−/−^) and DKO (PPARγ2^−/−^ L-PGDS ^−/−^) were ground on liquid nitrogen and homogenised in 15% (v/v) methanol in water, followed by the addition of internal standards 12-HETE-d8 and PGB_2_-*d*4 (40 ng) (Cayman Chemicals,Ann Arbor, MI, USA). The resulting solutions were then acidified with 0.1 M hydrochloric acid to pH 3.0 and immediately applied to pre-conditioned SPE cartridges (C18-E, 500 mg, 6 mL) from Phenomenex (Macclesfield, UK).The cartridges were washed with 15% (v/v) methanol in water (20 ml) followed by 20 ml of water (20 ml) and hexane (10 ml); the lipid mediators were eluted in methyl formate (15 ml). The organic solvent was evaporated using a fine stream of nitrogen and the remaining residue was re-dissolved in ethanol (100 µl) stored at –20°C awaiting analysis [24].

### LC-MS/MS Analysis of Eicosanoids

Chromatographic analyses were performed using Accela UHPLC (Thermo Scientific, Hemel Hempstead, UK) and Acquity UPLC systems (Waters, Hertsfordshire, UK). The bioactive lipids were separated on a C_18_ reversed-phase (RP) LC column (Phenomenex Luna, 3 µm particles, 150×2 mm) using a linear mobile phase gradient (A, 0.02% glacial acetic acid in water; B, 0.02% glacial acetic acid in acetonitrile). Mass spectrometry analyses were carried out on LTQ Velos (Thermo, Hemel Hempstead, UK) linear ion trap (LIT)-orbitrap and QTRAP 4000 (AB Sciex, Concord, ON) quadrupole-linear ion trap (QqLIT) mass spectrometers [24]. Eicosanoids analyzed in male C57Bl/6 mice of 5 months of age, n = 8 per group.

### Shotgun Lipidomic Analysis of Liver

Approximately 5 mg of liver, which was frozen directly in liquid N_2_, were weighed and liver tissue was diluted with 50 µl of PBS buffer and WAT was diluted with 150 µl of methanol and vertexed for 2 min. All samples were spiked with an internal standard (10 µl for liver) [25]. Lipids were extracted from the liver samples with 200 µl of chloroform:Methanol (2∶1) solvent. After vortexing for 2 min and incubating for 1 h for liver samples at room temperature, the lower layer (approximately 100 µl for liver samples) was separated by centrifugation at 10,000 rpm for 3 min at room temperature. Ten microliters of labelled standard mixture (three stable isotope-labeled reference compounds) for liver samples was added to the lipid extracts and 0.5 - 1.0 µl injection was used for LC/MS analysis. Sample order for analysis was established by randomization. Lipid extracts were analyzed on a Q-ToF Premier mass spectrometer (Waters) combined with an Acquity Ultra Performance Liquid chromatography (UPLC/MS). Mass spectrometry was carried out on Q-Tof Premier (Waters, Inc.) run in ESI+ mode. The data was collected over the mass range of m/z 300–1200 with scan duration of 0.2 sec. The source temperature was set at 120°C and nitrogen was used as desolvation gas (800 L/h) at 250°C. The voltages of the sampling cone and capillary were 39 V and 3.2 kV, respectively. Reserpine (50 µg/L) was used as the lock spray reference compound (5 µl/min; 10 sec scan frequency). The obtained data was converted into netCDF file format using Dbridge software from MassLynx (Waters, Inc.). The converted data was processed using MZmine software version 0.60 [26]. Lipids were identified based on their retention time (RT) and mass to charge ratio (MZ) using our in-house built lipid database as previously described [25]. All the identified lipids were quantified by normalizing with corresponding internal standards. Liver lipids analyzed in male C57Bl/6 mice of 5 months of age, n = 8 per group.

### Statistics

Results were expressed as mean ± SEM. Statistical analysis was performed using SPSS 17.0 (IBM, New York). Pair wise comparisons were carried out by students t-test. For data with more than two groups ANOVA was carried out followed by Tukeys Post-Hoc Test.

## Results

### Lack of PPARγ2 Results in Increased *L-PGDS* mRNA Expression Selectively in BAT and Subcutaneous WAT

**Figure 1:**
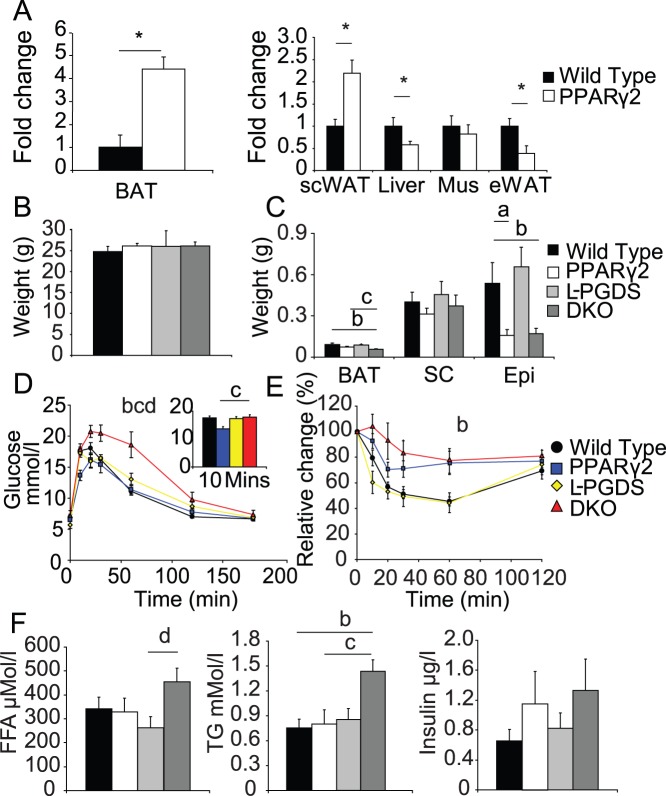
Regulation and function of L-PGDS in models of altered PPARγ activity. A) *L-PGDS* mRNA levels in the major metabolic tissues of *PPARγ2* KO mice. B) Body weights of all four genotypes from the *L-PGDS* x *PPARγ2* (DKO) double cross. C) DKO mice have reduced BAT mass compared to other genotypes. Both *PPARγ2* KO and DKO mice have reduced epididymal fat mass. D) DKO mice have impaired glucose tolerance compared to any other genotype. Statistics performed on area-of-curve. (Box, AOC 0–10 minutes). E) DKO mice are more insulin resistant than WT mice Statistics performed on area- of-curve. F) Serum Free fatty acid, triglyceride and insulin levels. All mice n = 8 per group C57Bl/6 males 4 months old, except mice undergoing ITT which were 4.5 months of age. *, P<0.05 **a**, P<0.05 WT vs PPARγ2, **b**, P<0.05 WT vs DKO **c**, P<0.05 PPARγ2 vs DKO, **d** P<0.05 L-PGDS vs DKO.

The sv129 *PPARγ2* KO mouse represents a model of impaired lipid handling but with apparently normal glucose tolerance. To try to identify mechanisms that may protect mice lacking *PPARγ2* from metabolic impairments we used microarrays to identify genes up regulated in BAT and WAT. L-PGDS was found to be highly up regulated in brown adipose tissue (data not shown). We confirmed these results by RT-PCR analysis on major metabolic tissues from PPARγ2 KO mice. *L-PGDS* expression was increased in BAT and scWAT from *PPARγ2* KO mice but down regulated in epididymal WAT and liver (Figure 1A).

### L-PGDS x PPARγ2 KO (DKO) Mice have Impaired Carbohydrate and Lipid Metabolism

To investigate if the up regulation of *L-PGDS* in BAT and scWAT was compensating for the absence of PPARγ2 function, a double KO mouse lacking both *L-PGDS* and *PPARγ2* (DKO) was generated. At 16 weeks of age body weights of chow fed mice appeared similar, irrespective of genotype (Figure 1B). However, the interscapular BAT mass of the DKO mice was significantly smaller than that of wild-type or *PPARγ2* KO mice (Figure 1C). Epididymal WAT mass was decreased in both the DKO and *PPARγ2* KO mice to an equal degree when compared to WT controls. In addition to alterations in adipose tissue mass, at 16 weeks of age, DKO mice had impaired glucose tolerance when compared to WT, *L-PGDS* or *PPARγ2* KO mice (Figure 1D). Subsequent insulin tolerance tests demonstrated more severe insulin resistance in the DKO mice, when compared to wild-type mice (Figure 1E). Serum biochemistry demonstrated that DKO mice exhibited elevated serum triglycerides - nearly double that of any other genotype. Furthermore DKO mice exhibited an increase in fed free-fatty acid levels over *L-PGDS* single KO mice (Figure 1F).

### L-PGDS x PPARγ2 KO Mice have Altered Levels of Markers of Brown Adipocyte Thermogenic Function

**Figure 2:**
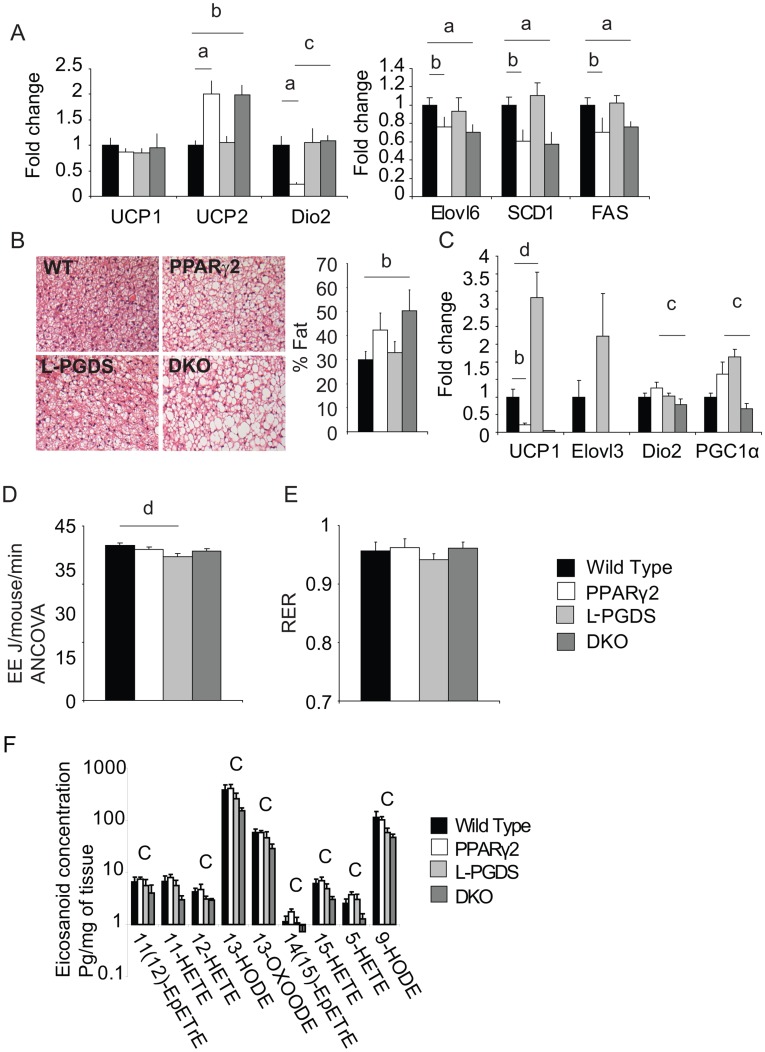
Energy expenditure and BAT function in DKO mice. A) Gene expression from interscapular BAT. B) Histological analysis of brown fat, analysis of lipid percentage area (right) and representative figures (left). C) Gene expression of thermogenic genes from subcutaneous WAT. D) Oxygen consumption in ml/mouse/min E) Respiratory exchange ratio (RER). All mice n = 8 per group C57Bl/6 males 4 months old. F) Levels of Eicosanoids detectable in the brown adipose tissue of all 4 genotypes n = 8 per group C57Bl/6 males 5 months old. **a**, P<0.05 WT vs PPARγ2, **b**, P<0.05 WT vs DKO **c**, P<0.05 PPARγ2 vs DKO, **d** P<0.05 L-PGDS vs DKO.

Alteration in BAT mass can occur as a consequence of reduced BAT development and/or function. However, gene expression analysis failed to show many DKO-dependent alterations in markers of BAT activation. Several PPARγ target genes were down regulated in response to PPARγ2 ablation (including *Elovl6, SCD1* and *FAS*), whereas *UCP2* was up regulated, however the changes in these genes occurred to an equal extent in *PPARγ2* KO mice and DKO mice (Figure 2A). Morphologically, interscapular BAT from DKO mice exhibited greater lipid content than WT mice (Figure 2B) but was not distinct from *PPARγ2* KO mice. Overall, these results demonstrated that at 24°C there was a very minor impact of a loss of L-PGDS on the function of canonical BAT depots in either WT or *PPARγ2* KO backgrounds.

In addition to traditional BAT depots, subcutaneous WAT has recently been shown to possess significant amounts of developmentally distinct brown adipocytes, albeit in scWAT the levels of brown adipocyte markers are far lower than in canonical BAT depots. In scWAT, *L-PGDS* KO mice exhibited a 4 fold increase in UCP1 expression. Conversely, *PPARγ2* KO mice exhibited a 5 fold reduction in *UCP1* when compared to WT, with DKO mice having similarly low *UCP1* expression levels to *PPARγ2* KO mice (Figure 2C). Conversely, *PGC1α* and *Deiodinase 2* were both down regulated in DKO mice compared to the *PPARγ2* KO mice.

To determine if reduced scWAT function impacted on energy balance, indirect calorimetry was performed on all 4 genotypes. At 22°C, *L-PGDS* KO mice had a reduction in metabolic rate compared to WT mice (Figure 2D), whereas DKO, *PPARγ2* KO and WT mice had similar metabolic rates. Analysis of RER showed no overall difference in RER between groups (Figure 2E). Overall the lack of changes in metabolic rate and RER between the *PPARγ2* KO and DKO mice suggested there was no impairment in metabolic rate or gross substrate utilisation on a whole organism level as a result of a loss of L-PGDS in the *PPARγ2* KO background.

### Subtle Alterations in Eicosanoid Levels in Brown Adipose Tissue are not Consistent with a Prostaglandin Synthase Role for L-PGDS

L-PGDS is capable of acting as both a secreted carrier of lipophylic molecules and as a prostaglandin synthase producing PGD_2_. To try to determine which role was important for the metabolic alterations seen in DKO mice we analysed eicosanoid levels by LC-MS/MS analysis. Several eicosanoid species were reduced in DKO mice compared to PPARγ2 KO mice (Figure 2F), however these were principally lipoxygenase products. In brown adipose tissue, none of the prostaglandins detected showed significant differences, however their levels were, below the limit for accurate quantification (data not shown). Overall, the lack of changes in cycloxygenase products and their extremely low levels suggest that, L-PGDS may affect BAT metabolism by acting as a lipid-carrying lipocalin rather than a prostaglandin synthase.

### DKO Mice have Markers of Altered Lipid Metabolism in WAT

The elevated circulating FFA levels present in the DKO mouse compared to the L-PGDS KO alone pointed to a potential alteration in adipose tissue function. To investigate this further, gene expression analysis was carried out on subcutaneous and epididymal white adipose tissue depots.

**Figure 3:**
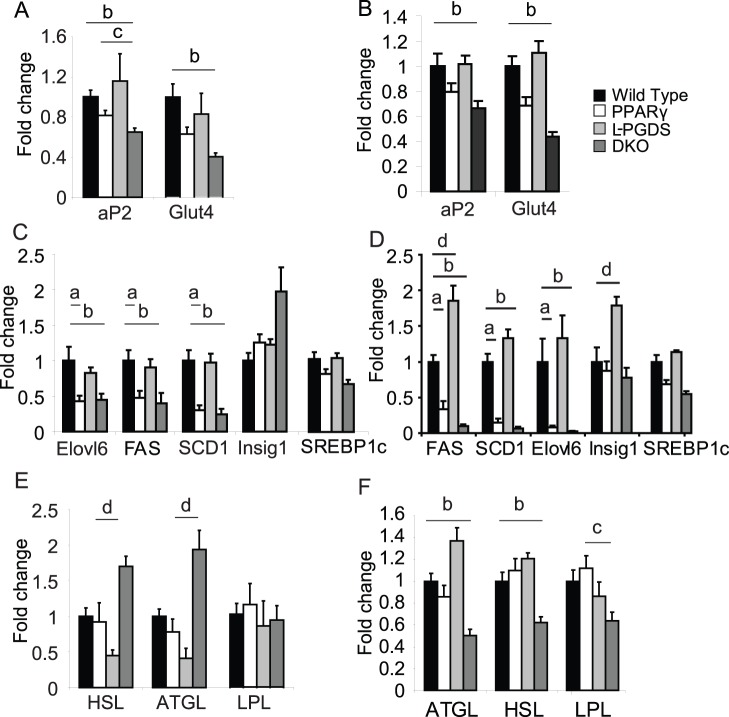
Gene expression from epididymal and subcutaneous adipose tissue depots. Expression of adipogenic markers from; A) epididymal white adipose tissue, B) subcutaneous white adipose tissue. Expression of lipogenic markers from; C) epididymal white adipose tissue, D) subcutaneous white adipose tissue. Expression of lipases from; E) epididymal white adipose tissue, F) subcutaneous white adipose tissue. All mice n = 8 per group C57Bl/6 males 4 months old. **a**, P<0.05 WT vs PPARγ2, **b**, P<0.05 WT vs DKO **c**, P<0.05 PPARγ2 vs DKO, **d** P<0.05 L-PGDS vs DKO.

Both DKO and PPARγ2 KO mice had reductions in the adipogenic marker aP2 compared to WT. DKO mice also had significantly lower levels of aP2 to PPARγ2 KO mice in epididymal WAT. Glut4 expression which can be related to both adipogenesis and insulin sensitivity was down regulated only in DKO mice when compared to WT (Figures 3 A and B).

Analysis of the *de novo* lipogenic program in subcutaneous and epididymal WAT revealed a general reduction in the expression of lipogenic genes in mice lacking *PPARγ2*, with significant reductions in FAS, SCD1 and ELOVL6 in both depots of *PPARγ2* KO and DKO mice (Figure 3C and D). Notably, the magnitude of the reduction in the expression of the *de novo* lipogenic program was much more substantial in scWAT than epididymal. Mice lacking *L-PGDS* alone exhibited the opposite pattern, with increases in *FAS and Insig1* expression exclusively in subcutaneous white adipose tissue (Figure 3D).

Most strikingly, analysis of lipase expression demonstrated a reciprocal regulation of HSL and ATGL between epididymal and subcutaneous adipose tissue depots. DKO mice had increased expression of ATGL and HSL compared to L-PGDS KO mice alone in epididymal white adipose tissue (Figure 3E). Conversely, in subcutaneous white adipose tissue the expression of ATGL and HSL was decreased in DKO mice compared to WT mice (Figure 3F). Of note, the pattern of the expression of HSL and ATGL in epididymal white adipose tissue was similar to the pattern of levels of FFA observed in serum (Figure 1F). LPL expression was unchanged in epididymal white adipose tissue, whereas it was down regulated in subcutaneous white adipose tissue of DKO mice when compared to WT (Figure 3F). Overall these data were suggestive of a greater impairment in subcutaneous adipose tissue function when compared to epididymal adipose tissue in terms of lipogenesis, lipid uptake and lipid release.

**Figure 4:**
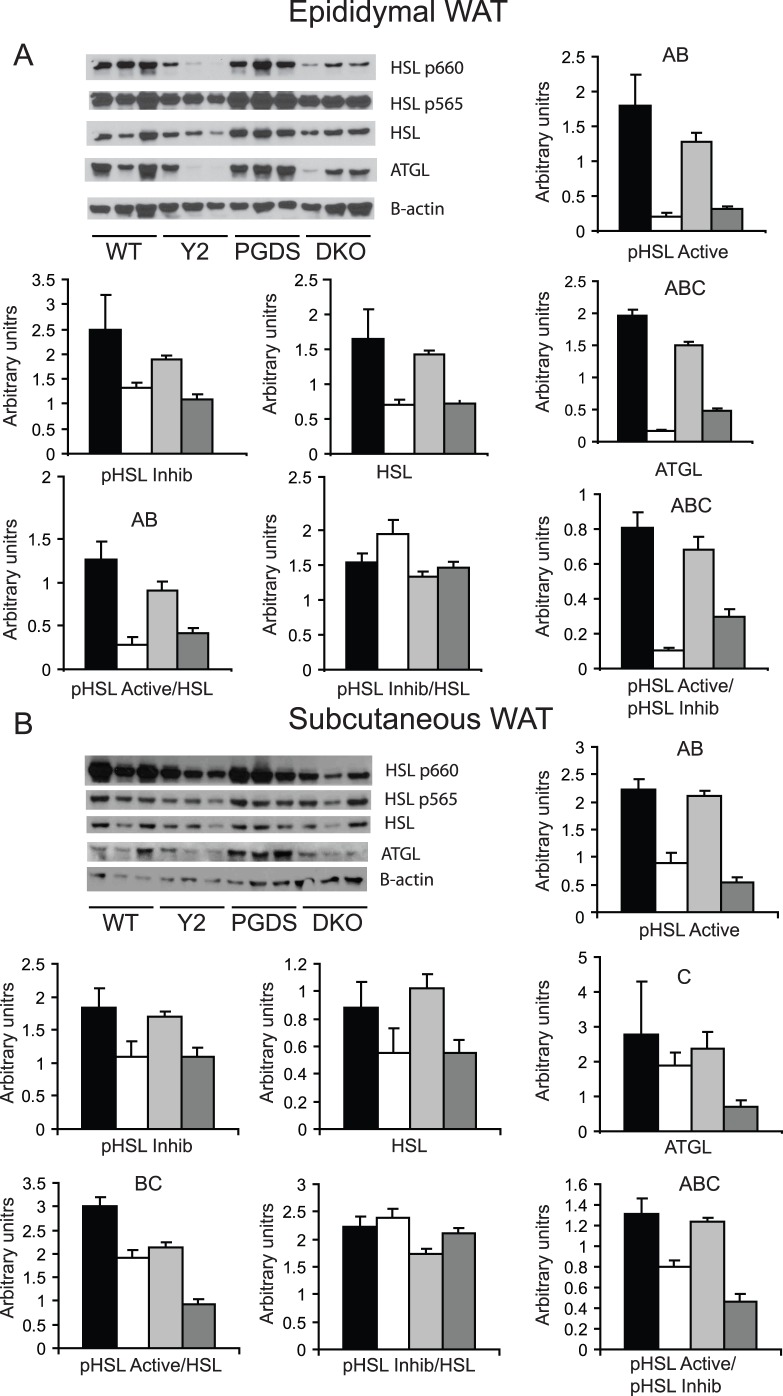
Regulation of lipolytic proteins in WAT depots. Western blots from A) epididymal and B) subcutaneous white adipose tissue. All quantifications based on densitometry and normalised to β-actin. Representative blots show N  = 3 mice. Quantification N  = 6 for each genotype for all genes except subcutaneous ATGL, N  = 3. **a**, P<0.05 WT vs PPARγ2, **b**, P<0.05 WT vs DKO **c**, P<0.05 PPARγ2 vs DKO.

To provide further evidence for alterations in adipose depot-specific lipid handling, we analysed protein levels of HSL and ATGL as well as the phosphorylation status of HSL as a surrogate of HSL activation [27]. In epididymal WAT, DKO mice had a significant increase in ATGL levels compared to PPARγ2 mice, but both PPARγ2 and DKO mice had lower ATGL levels than WT (Figure 4A). In subcutaneous WAT, DKO mice had significantly lower ATGL protein expression than PPARγ2 KO or WT mice (Figure 4B). In terms of HSL, the absolute protein levels bore relatively little relation to the mRNA levels, with total HSL levels tending to be lower in the PPARγ2 and DKO mice compared to wild-type, but no different to each other in both subcutaneous and epididymal white fat. However, analysis of the S660 (activatory) and S565 (inhibitory) phosphorylation sites on HSL revealed that in epididymal WAT, mice DKO mice had a significantly increased ratio of activatory to inhibitory phosphorylation levels compared to PPARγ2 KO mice. In subcutaneous WAT the opposite was observed, with DKO mice having reduced levels of activatory HSL phosphorylation and a decreased activatory to inhibitory phosphorylation ratio compared to PPARγ2 KO mice.

### Altered Lipid Metabolism in DKO Mice is Associated with Markers of Increased Insulin Resistance in Epididymal WAT

**Figure 5:**
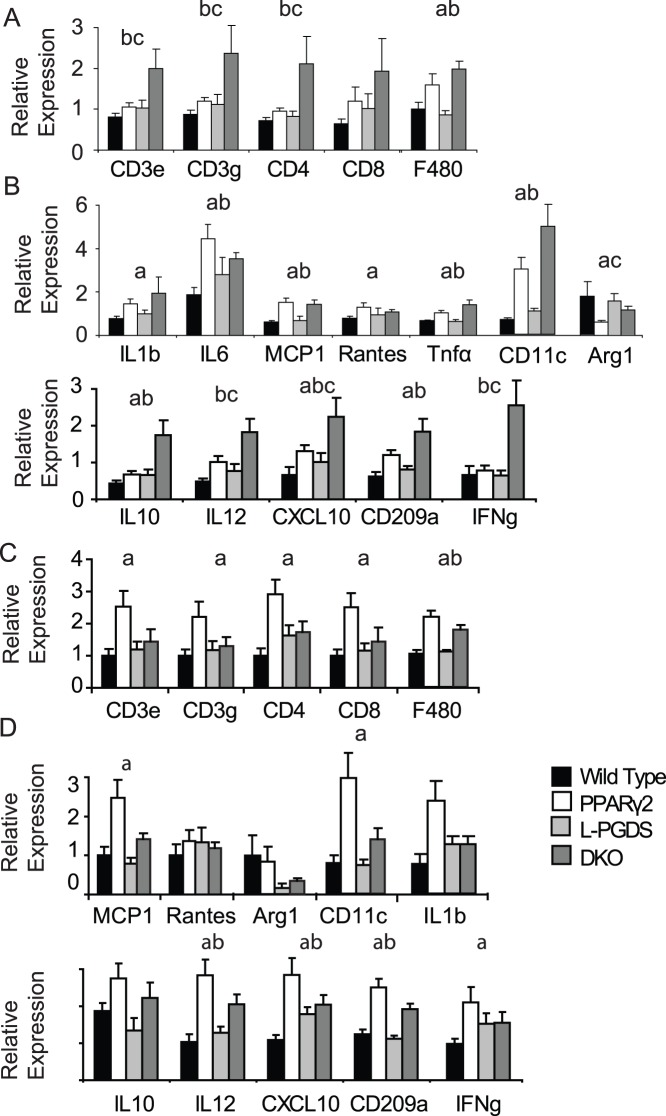
Expression of inflammatory markers in WAT depots. Gene expression from epididymal white adipose tissue of; A) markers of different macrophage and T-cell subtypes B) cytokines and chemokines and markers of macrophage polarisation. Gene expression from subcutaneous white adipose tissue of; C) markers of different macrophage and T-cell subtypes D) cytokines, chemokines and markers of macrophage polarisation All mice n = 8 per group C57Bl/6 males 4 months old. **a**, P<0.05 WT vs PPARγ2, **b**, P<0.05 WT vs DKO **c**, P<0.05 PPARγ2 vs DKO, **d** P<0.05 L-PGDS vs DKO.

Overall, epididymal WAT from DKO mice had a protein expression and phosphorylation pattern consistent with increased rates of lipolysis compared to PPARγ2 KO mice, which contrasted with the situation in scWAT the reverse pattern was seen. In humans, intra-abdominal fat has been shown to have both increased lipolytic rate and to be more inflamed than subcutaneous depots. We next investigated if the alterations in lipid metabolism observed in the DKO mouse were associated with inflammation in epididymal WAT. Analysis of inflammatory markers in epididymal WAT demonstrated a significant increase in the mRNA levels of T-cell markers *CD3e*, *CD3g* and *CD4* in DKO mice over WT and PPARγ2 KO mice (Figure 5A). Expression of macrophage markers and cytokines demonstrated a general elevation in mice lacking *PPARγ2*; however there was a trend towards higher levels of pro-inflammatory markers (*CD11c*, *TNFα*) in DKO mice compared to PPARγ2 KO alone and significant increases in *IFNγ* and *IL-12* (Figure 5B). In subcutaneous adipose tissue the general trend was reversed, with increased expression of T-cell markers *CD3e*, *CD3g* and *CD4* in *PPARγ2* KO mice, but not in DKO mice when compared to WT (Figure 5C). While F4/80 levels, a marker of macrophage infiltration, were similar between PPARγ2 and DKO mice in both epididymal and subcutaneous white adipose tissue, levels of the M1 polarisation marker *CD11c*, which has been associated with a worsened adipose tissue metabolic profile and levels of *IFNγ* were only induced in *PPARγ2* KO mice compared to WT mice (Figure 5D).

### Increases in the *de novo* Lipogenic Program in Liver from DKO Mice

**Figure 6:**
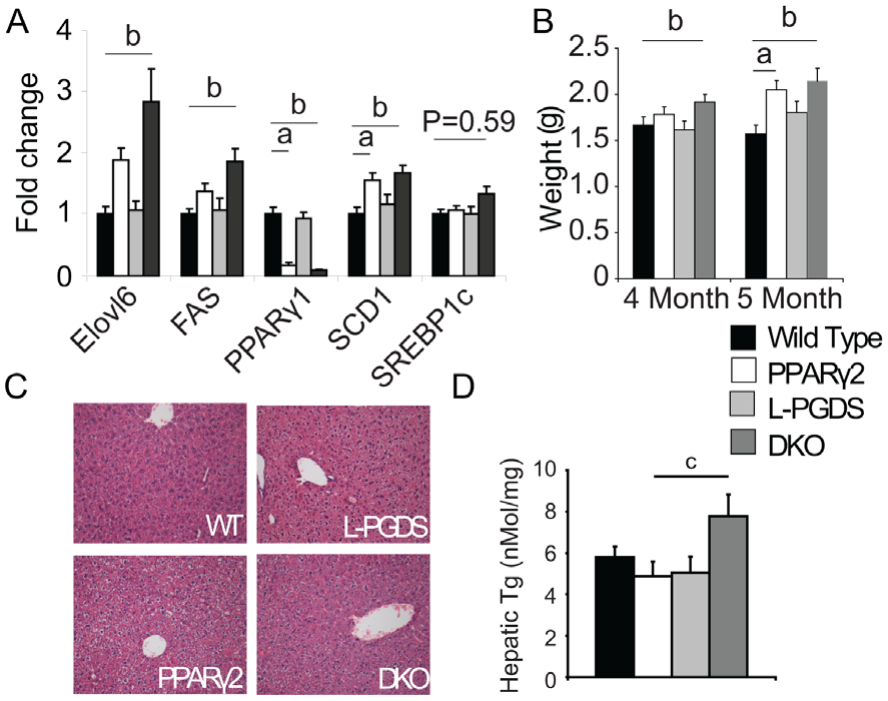
Gene expression and lipid levels in liver. A) DKO mice have increased expression of genes involved in hepatic lipogenesis B) DKO mice have accelerated hepatomegaly C) DKO mice do not have overt hepatosteatosis. D) DKO mice have elevated hepatic TG levels. All mice n = 8 per group C57Bl/6 males 5 months old. **a**, P<0.05 WT vs PPARγ2, **b**, P<0.05 WT vs DKO **c**, P<0.05 PPARγ2 vs DKO, **d** P<0.05 L-PGDS vs DKO.

While the changes in FFA levels where most likely to have come from alterations in adipose tissue function, the alterations in TG levels could either have been down to reduced TG clearance or due to increased de novo synthesis in liver. Gene expression analysis of liver showed that when compared to other genotypes DKO mice had a significant increase in expression of *FAS* and *ELOVL6* compared to wild-type mice (Figure 6A) they also tended (P = 0.059) to have increased expression of *SREBP1c*. Consistent with markers of increased rates of lipid synthesis, livers from DKO mice were significantly heavier at 4 months than wild-type mice (Figure 6B). Although relatively small, the changes in liver mass between the DKO and wild-type were significant in two independent groups. Although there was no obvious change in hepatic steatosis as assayed by histology (Figure 6C), DKO mice had a 25% increase in hepatic TG concentration compared to PPARγ2 KO mice (Figure 6D), supportive of the elevated *de novo* lipogenic program and the elevated serum TG levels. Finally, lipidomic analysis of the livers of DKO mice revealed, when compared to PPARγ2 KO mice, an up regulation in the levels of lysophosphatidylcholines, which have been associated with insulin resistance (Figure S1).

## Discussion

We previously reported that mice lacking PPARγ2 had an unexpectedly normal metabolic phenotype [1]. In this study we have demonstrated that *L-PGDS* was up-regulated selectively in BAT and subcutaneous white adipose tissue and that L-PGDS in part compensated for a loss of PPARγ2 function. Simultaneous loss of PPARγ2 and L-PGDS synergistically caused glucose intolerance. Additionally mice lacking PPARγ2 and L-PGDS had elevated serum triglyceride compared to any other genotype. These biochemical parameters were associated with depot-specific changes in expression of lipases, HSL phosphorylation status and expression of inflammatory markers in white adipose tissue. They were also associated with an increased expression of the *de novo* lipogenic program in liver in DKO mice when compared to wild-type mice.

Loss of PPARγ2 resulted in an increase in *L-PGDS* expression in brown and subcutaneous WAT but a decrease in *L-PGDS* expression in epididymal WAT and liver. Given the adipose depot-specific profile of *L-PGDS* regulation, we expected to see the most dramatic effects of a simultaneous loss of both L-PGDS and PPARγ2 in BAT and subcutaneous WAT. Although there were relatively modest changes in gene expression in the inter-scapular BAT of double knock-out mice, the subcutaneous WAT depot showed much more dramatic alterations. In subcutaneous WAT, in terms of gene expression, loss of PPARγ2 led to an almost complete switching off of both the thermogenic program and the *de novo* lipogenic program, regardless of the presence of L-PGDS. However, the loss of L-PGDS and PPARγ2 together resulted in a reduction in *HSL*, *ATGL* and *LPL* expression in subcutaneous WAT. These changes were qualitatively mirrored at the protein level, with a reduction in the ratio of activating and inhibitory phosphorylation levels on the lipase HSL of DKO mice compared to PPARγ2 KO mice. Added to the individual effect of a loss of PPARγ2 on *de novo* lipogenesis and thermogenesis, these results were consistent with a general impairment in subcutaneous adipose tissue function in DKO mice, suggesting this tissue may have become metabolically inert.

While the alterations in carbohydrate metabolism and circulating TG levels in the DKO mice were consistent with alterations in subcutaneous lipid handling genes, the lack of a reduction in metabolic rate and increase in circulating FFA levels suggested that the overall fuel availability was not compromised. Indeed, the loss of appropriate lipid release and synthesis of fatty acids in subcutaneous WAT appeared to be over-compensated for by increased markers of lipogenesis in liver and by increased markers of lipid release from epididymal WAT, on mRNA, protein and phosphoprotein levels. On a protein levels the same pattern was not as clear, as both PPARγ2 KO and DKO mice had reduced levels of ATGL and HSL, as well as reduced levels of HSL phosphorylation, however the DKO mice had significantly elevated levels of ATGL protein and an increased pHSLactive/pHSLinhib ratio compared to PPARγ2 KO mice alone. An important caveat is that while these markers support the concept of depot-specific alterations in lipolysis, direct measurements of lipolysis would be required to further confirm this finding.

The markers of increased lipid release in epididymal WAT were associated with multiple markers of increased inflammation in this tissue. In humans, mesenteric adipose tissue has both higher rates of lipolysis [9] and is more inflamed than subcutaneous adipose depots in obesity [28], [29]. Consistent with these observations, a recent study has demonstrated elevated lipolysis from adipose tissue can directly promote macrophage infiltration [30]. Furthermore, at least one report has demonstrated that L-PGDS expression is lower in mesenteric adipose tissue than subcutaneous adipose tissue depots [19]. We hypothesise that in the context of an underlying impairment in adipose tissue function caused by reduced PPARγ function, loss of L-PGDS reduced subcutaneous adipose tissue function further in terms of both uptake and release of lipid and resulted in an over-reliance on intra-abdominal adipose tissue, associated with elevated inflammation.

The changes in gene expression and lipid levels in liver, despite the lack of a primary change in L-PGDS expression, may be secondary to alterations in adipose tissue lipid handling observed in the DKO mice. The adipose tissue expandability hypothesis has principally been focused on the concept that a reduction in adipose tissue mass leads to ectopic deposition of lipid in non adipose organs, [31]. More recently, evidence from mice and humans suggests that adipose tissue dysfunction in terms of lipid uptake and release, may be the most important factor that leads to ectopic lipid deposition [32] [27]. The accumulation of triglyceride in liver and the elevated levels of the potentially lipotoxic species lysophosphatidylcholine [33]–[34] in the DKO mice when compared to PPARγ2 KO mice occur despite no differences in fat pad masses between these animals. Despite similar fat pad masses between PPARγ2 and DKO mice, DKO mice had increased ATGL levels and a pro-activation HSL-phosphorylation pattern in epididymal white adipose tissue. The changes in ATGL and HSL were reversed in subcutaneous adipose tissue. Overall, these data suggest that functional changes primarily occurring in adipose tissue depots could be mediating secondary changes in liver via lipotoxic process.

An important teleological question is why L-PGDS, an apparently positive factor for metabolic health in this setting, is elevated in response to ablation of PPARγ2 another apparently positive factor. We and others have put forward the concept of allostasis, which suggests that complex systems, such as mice, have multiple parallel systems which perform similar though non-identical roles. When one system is ablated, other systems are able to partially compensate for the loss of the primary system, albeit usually at some cost to fitness [13]. Mechanistically, how L-PGDS acts to compensate for a loss of PPARγ2 remains unresolved. Measurements of eicosanoids isolated from BAT from WT, *L-PGDS* KO, *PPAR*γ*2* KO and DKO mice revealed some subtle alterations in lipoxygenase products. However, cyclooxygenase products, including PGD_2_ itself, were detected at levels too low for accurate quantification and showed no significant differences. Overall these data suggest that L-PGDS may act as a lipocalin rather than as a prostaglandin synthase [15], [35], [36]. What lipid-signalling molecule L-PGDS is bound to, or even if L-PGDS itself acts directly as a signalling molecule binding to an as yet unidentified surface receptor, remains to be determined. The ability of L-PGDS to act as a circulating factor could also be responsible for the relatively strong phenotype observed in liver, despite the down regulation in L-PGDS expression.

Overall, we conclude that L-PGDS compensates in part for a loss of PPARγ2 in vivo. Undoubtedly the phenotype is complex, containing aspects of both L-PGDS and PPARγ2 ablations alone, as well as additive and synergistic effects of the loss of both molecules. However, in the context of a PPARγ2-null background L-PGDS is in part necessary to allow maintenance of appropriate lipid handling and glucose tolerance. Gene and protein expression analysis of white adipose tissue depots suggests that L-PGDS may regulate the relative contribution of subcutaneous and abdominal white adipose tissue depots in the control of lipid uptake and release.

## Supporting Information

Figure S1 **Levels of Lysophosphatidylcholine species in liver.** A) DKO mice have elevated levels of Lysophosphatidylcholine species in liver. All mice n = 8 per group C57Bl/6 males 5 months old. C, P<0.05 PPARγ2 vs DKO.
(EPS)
